# The role of WNT5A and Ror2 in peritoneal membrane injury

**DOI:** 10.1111/jcmm.15034

**Published:** 2020-02-13

**Authors:** Manreet Padwal, Limin Liu, Peter J. Margetts

**Affiliations:** ^1^ Department of Medicine McMaster University Hamilton ON Canada

**Keywords:** angiogenesis, epithelial to mesenchymal transition, fibrosis, peritoneal dialysis, Ror2, TGFB, VEGF, WNT/Bcatenin, WNT/B‐catenin signalling, WNT5A

## Abstract

Patients on peritoneal dialysis are at risk of developing peritoneal fibrosis and angiogenesis, which can lead to dysfunction of the peritoneal membrane. Recent evidence has identified cross‐talk between transforming growth factor beta (TGFB) and the WNT/β‐catenin pathway to induce fibrosis and angiogenesis. Limited evidence exists describing the role of non‐canonical WNT signalling in peritoneal membrane injury. Non‐canonical WNT5A is suggested to have different effects depending on the receptor environment. WNT5A has been implicated in antagonizing canonical WNT/β‐catenin signalling in the presence of receptor tyrosine kinase‐like orphan receptor (Ror2). We co‐expressed TGFB and WNT5A using adenovirus and examined its role in the development of peritoneal fibrosis and angiogenesis. Treatment of mouse peritoneum with AdWNT5A decreased the submesothelial thickening and angiogenesis induced by AdTGFB. WNT5A appeared to block WNT/β‐catenin signalling by inhibiting phosphorylation of glycogen synthase kinase 3 beta (GSK3B) and reducing levels of total β‐catenin and target proteins. To examine the function of Ror2, we silenced Ror2 in a human mesothelial cell line. We treated cells with AdWNT5A and observed a significant increase in fibronectin compared with AdWNT5A alone. We also analysed fibronectin and vascular endothelial growth factor (VEGF) in a TGFB model of mesothelial cell injury. Both fibronectin and VEGF were significantly increased in response to Ror2 silencing when cells were exposed to TGFB. Our results suggest that WNT5A inhibits peritoneal injury and this is associated with a decrease in WNT/β‐catenin signalling. In human mesothelial cells, Ror2 is involved in regulating levels of fibronectin and VEGF.

## 1 INTRODUCTION

Peritoneal dialysis (PD) is a common treatment for patients with chronic kidney disease or end‐stage renal disease.^1^ The healthy peritoneum is a semipermeable membrane consisting of a superficial mesothelial layer, a basement membrane and a thin submesothelial zone.^2^, ^3^ During PD, hyperosmotic dialysis fluid is used to generate a concentration gradient, which drives diffusion of fluids and dissolved substance across the peritoneal membrane. This results in the removal of wastes and toxins from the blood.^4^ Continuous time spent on PD results in two major structural changes in the peritoneum including thickening of the submesothelium and expansion of the membrane vasculature.^1^, ^2^ Studies have also documented an increase in peritoneal membrane solute transport over time.^5^ The increase in angiogenesis is associated with an increase in solute transport rate and a decline in the ultrafiltration capacity.^3^, ^4^, ^6^ Decreased ultrafiltration capacity, combined with loss of residual renal function, may predict for subclinical volume expansion, which is associated with inflammation and cardiac hypertrophy.^7^

Transforming growth factor beta (TGFB) is a predominant cytokine involved in the initiation and progression of peritoneal fibrosis.^3^, ^4^ Mesothelial cells secrete TGFB in response to injury from dialysis solutions and infection.^4^ TGFB has been observed to induce changes in peritoneal structure including thickening of the submesothelial zone and angiogenesis. In conjunction with TGFB, the WNT signalling family has also been reported to play an important role in the initiation and progression of fibrosis in different organ systems.^8^, ^9^ WNT signalling can be separated into canonical signalling and non‐canonical signalling; canonical WNT signalling utilizes a β‐catenin dependent pathway. Canonical WNT signalling has been observed to interact with the TGFB pathway to potentiate fibrosis in the kidney,^8^, ^9^ liver,^10^ lungs 11 and skin.^12^ The WNT family of proteins can be loosely separated into canonical vs non‐canonical WNT ligands, and WNT5A is considered a prototypical non‐canonical WNT protein.^13^

The role of WNT signalling in peritoneal membrane injury has been recently clarified. We examined the interaction between TGFB and WNT signalling in the development of peritoneal fibrosis and angiogenesis. We observed WNT/β‐catenin signalling to be active during TGFB‐induced peritoneal membrane injury, and our results suggest cross‐talk with the TGFB pathway during the development of injury.^14^ We used Dickkopf‐related protein (DKK‐1) to inhibit WNT/β‐catenin signalling and observed a decrease in both angiogenesis and also epithelial to mesenchymal transition (EMT).^14^ In a similar study of dialysis infused peritoneal fibrosis, β‐catenin was shown to induce EMT.^15^ Our results concur with reports of WNT signalling in other fibrotic diseases.^9^, ^10^, ^11^, ^16^, ^17^, ^18^

The majority of studies in fibrotic diseases have focused on the role of β‐catenin dependent signalling. There are few studies on the pathological role of non‐canonical WNT5A in fibrosis. Physiologically, WNT5A has been observed to regulate cell polarity and intracellular calcium levels through the planar cell polarity pathway (PCP) and calmodulin‐dependent protein kinase II pathway, respectively.^19^ During acute wound repair, the fibroblasts deposit extracellular matrix and myofibroblasts migrate to mediate contraction of the wound. In vitro studies of wound healing have demonstrated that WNT5A induces cell migration and assists in wound closure suggesting a potential role in preventing fibrosis.^13^, ^20^, ^21^ A few studies have revealed elevated levels of WNT5A in fibrosis of the liver 22 and in patients with idiopathic pulmonary fibrosis and acute lung injury.^23^, ^24^, ^25^ Although these studies shed some light on the gene expression profile of WNT5A, further investigation is required to understand why WNT5A is up‐regulated during wound healing and fibrosis and if it is exerting a positive or negative effect. Emerging studies in fibrosis suggest that WNT5A contributes to progression of fibrosis by inducing fibroblast proliferation^26^, ^27^, ^28^ and by inducing matrix production.^29^, ^30^ In the studies that have analysed receptor environment, the Frizzled (FZD) family of receptors has been involved in mediating the injurious effects of WNT5A.^31^

In studies of tumorigenesis, WNT5A has been reported to have opposing effects. WNT5A has been shown to work as a tumour suppressor in specific types of cancer.^32^, ^33^, ^34^ In contrast, it has been associated with the progression of many cancers and its expression is strongly associated with invasion and metastasis.^21^, ^35^ Moreover, in the process of EMT, WNT5A has been reported to suppress 33 but also induce EMT during tumour development.^36^ WNT5A has also been described to regulate vascular development during normal embryogenesis playing a role in endothelial cell differentiation as well as during tumour development.^37^ Overall, the adverse effects of WNT5A seem to occur in the presence of FZD^36^ and we suggest that the role of WNT5A in cellular function may be depend on the nature of the WNT receptors present on the cell surface.

WNT5A has been observed to mediate signalling through receptor tyrosine kinase‐like orphan receptor 2 (Ror2) and through FZD 3, 4, 5 and lipoprotein receptor‐related proteins (LRP) 5 and 6. WNT5A and Ror2 have been demonstrated to interact physically and also functionally by activating the PCP pathway to regulate the cytoskeleton during embryogenesis.^38^ Ror2 can also interact with several canonical WNT proteins. In addition to this, accumulating evidence suggests that WNT5A can have both antagonistic and agonistic effects on the WNT/β‐catenin pathway based on the receptor it is signalling through.^13^, ^39^ Mikels et al^13^, ^40^ investigated the signalling of WNT5A in the presence of two different receptors, Ror2 or FZD4. Ror2 was observed to interact with WNT5A to inhibit the WNT/β‐catenin pathway. However, in the presence of FZD4, WNT5A potentiated WNT/β‐catenin signalling.^13^, ^40^, ^41^

The role of WNT5A and Ror2 in peritoneal membrane injury has not been explored. Aberrant WNT/β‐catenin signalling has been observed to contribute to progressive fibrosis in several different organs.^8^, ^10^, ^42^ We suggest that WNT5A may block canonical WNT signalling and reduce injury. In our study, we examined the function of WNT5A in TGFB‐induced peritoneal membrane injury. We found that WNT5A reduced submesothelial thickening and blood vessel density in the mouse peritoneum. Further, we observed decreased amounts of total β‐catenin and phosphorylated glycogen synthase kinase 3 beta (pGSK3β) suggesting inhibition of the WNT/β‐catenin pathway. Next, we explored the function of Ror2 more closely by silencing Ror2 in the setting of TGFB‐induced mesothelial cell injury. We found that Ror2 is important in regulating levels of VEGF and fibronectin in these cells.

## 2 METHODS

### 2.1 Recombinant adenovirus

The construction of the adenovirus vector AdTGFB has been previously described. AdTGFB expresses constitutively active TGFB1.^43^ AdTGFB was created with TGFB1 cDNA mutated at residues 223 and 225, so that the transgene product does not bind to the latency‐associated protein and is therefore biologically active. A null adenovirus (AdDL) was used as a control. AdWNT5A expressing murine WNT5a was a kind gift from Dr Tong Chuan He. Amplification and purification of adenoviruses was performed using CsCl gradient centrifugation as previously described. Titration of adenovirus was completed using 293 cells as previously described.^43^ HK‐2 cells were treated with AdWNT5A to confirm successful adenoviral‐mediated gene transfer of WNT5A (figure not shown).

### 2.2 Animal experiments

All animal studies were performed according to the Canadian Council on Animal Care guidelines. All animal studies were approved by McMaster University's Animal Research Ethics Board (AREB). Animals were housed under specific pathogen‐free conditions and were administered treatments in their home cage. Mice (C57BL/6, 5‐6 weeks; Harlan, Indianapolis, IN, USA) were injected intrapertioneally with 1 × 10^8^ plaque‐forming units (pfu) of first generation AdDL (n = 3 animals/group), AdWNT5A (n = 5 animal/group), AdTGFB + AdDL (n = 5 animals/group) or AdWNT5A + AdTGFB (n = 4 animals/group). AdTGFB or AdWNT5A was diluted to 100 mL in phosphate‐buffered saline. Animals were infused with a peritoneal dwell of 4.25% glucose dialysis solution an hour prior to kill. Animals were anesthetized with isoflurane (MTC Pharmaceuticals, Cambridge, ON, Canada) and killed at day 10 using anaesthesia and exsanguination.

At sacrifice, the entire anterior abdominal wall was then removed and divided into two sections. The lower section was placed in 10% formalin for histological analysis, and the upper section was placed in 1 mL Easy‐blue™ reagent (Froggabio) for RNA isolation. Omental tissue was taken and frozen in liquid nitrogen for protein analysis.

### 2.3 Cell culture experiments

Human mesothelial cells (MET5A) (Cat No. CRL‐9444, ATCC) were cultured in M199 media (Sigma‐Aldrich Canada Ltd). In the first experiment, human MET5A cells were transfected with siRNA against Ror2 (Stealth, Invitrogen) or a non‐targetting siRNA (Fisher Scientific) for 16 hours. Following this, serum‐starved cells were treated with AdWNT5A (10 MOI) for 24 hours. In the following experiment, human MET5A cells were transfected with small interfering RNA (siRNA) against Ror2 (Stealth, Invitrogen) or non‐targetting siRNA (Fisher Scientific). Twenty‐four hours after transfection, serum‐starved cells were treated with rTGFB (R&D Systems) at 2.5 ng/mL or PBS for 8 hours. Cells were harvested to collect supernatant, protein and RNA.

### 2.4 Histology

Tissue samples were fixed in a sufficient amount of 10% neutral‐buffered formalin for 48 hours, followed by 70% ethanol. The tissue samples were processed, paraffin embedded and cut in 5‐mm sections. Sections were stained with Masson's trichrome. Sections were also stained using vonWillebrand Factor antibody/Factor VIII (Dako) to measure blood vessel density. Five images were taken per stained section from each animal. Image J was used to quantify blood vessel density and thickness of the submesothelial zone.

### 2.5 Immunofluorescence

Immunofluorescence was performed by using a monoclonal mouse anti‐human α smooth muscle actin (α‐SMA) antibody (1:50) (Dako) with a Texas Red goat antimouse secondary antibody (1:100) (Molecular Probes, Life Technologies) followed by a FITC‐labelled monoclonal mouse antibody against pan‐cytokeratin (1:50) (Sigma‐Aldrich). Slides were mounted with nuclear Dapi (Vector Laboratories). Quantification of dual‐labelled cells in the mesothelial and submesothelial zones was completed using ImageJ software (National Institutes of Health). Cells co‐expressing α‐SMA and pan‐cytokeratin were classified as dual labelled. Dual‐labelled cells were identified in the mesothelial cell layer and in the submesothelial cell layer from five high‐power fields from each section using a blinded approach.

### 2.6 Protein analysis

Protein was extracted from frozen omental tissue by homogenization in a standard lysis buffer with proteinase inhibitors. Equal amounts of the protein were run on a 8% or 10% SDS‐PAGE gel, transferred to a membrane, and probed with VEGF (1:1000, Millipore, Etobicoke, ON, Canada), pGSK3β (1:1000, Cell Signalling), GSK3β (1:1000, Cell Signalling, Whitby, ON, Canada), total b‐catenin (1:1000, Cell Signalling), WNT5A (1:250, R&D, Toronto, ON, Canada), Ror2 (1:200, Santa Cruz, CA, USA), Fibronectin (1:1000, Sigma‐Aldrich), β‐actin (1:1000, Sigma‐ Aldrich) and α‐Tubulin (1:1000, Sigma‐Aldrich). Western blot analysis was completed in Image J using the Gel Analyzer Program.

Concentration of human VEGF was measured in cell culture supernatant using a standard ELISA kit (Sigma‐Aldrich Canada Ltd) according to the manufacturer's instructions.

### 2.7 Gene expression analysis

RNA was extracted from MET5A cells according to manufacturer's instructions (Invitrogen). The concentration of RNA obtained was measured by nanodrop. 1 μg of RNA obtained was reverse transcribed by RT‐PCR. Gene expression of VEGF was determined by 7500 Real Time PCR (Applied Biosystems). Samples were run in duplicate and normalized to 18s housekeeping gene. A standard curve generated from pooled mRNA samples was used for comparative quantification. Results are reported relative to 18s RNA.

### 2.8 Statistical analysis

Differences between two groups were evaluated using *t* test. Differences between multiple groups were assessed using ANOVA with Tukey's post hoc analysis. Analysis was carried out using SPSS v 22.0 (IBM).

## 3 RESULTS

### 3.1 WNT5A inhibits TGFB‐induced fibrosis and angiogenesis

**Figure 1:**
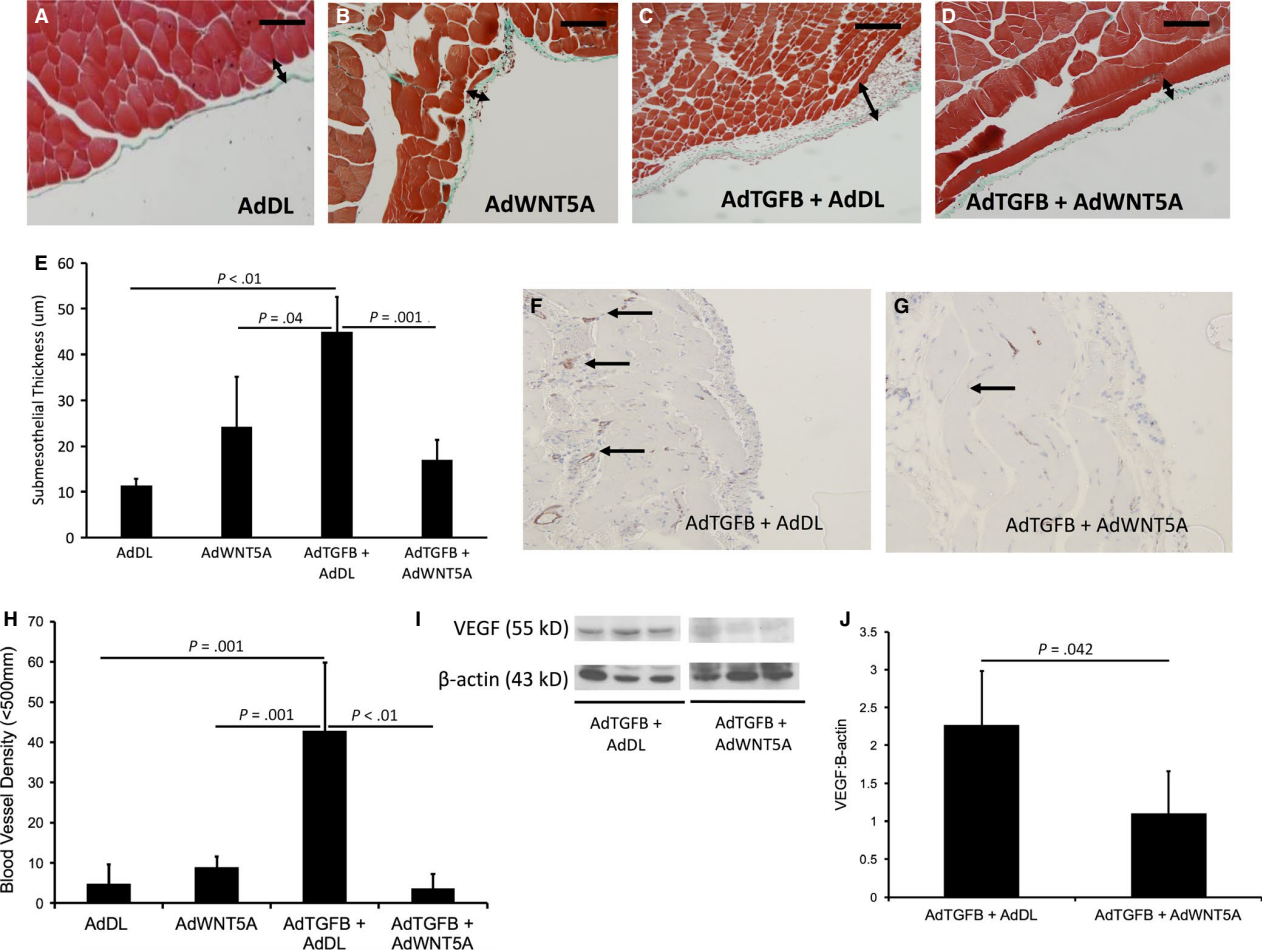
A‐D, Trichrome‐stained sections of C57Bl/6 mouse peritoneum after adenoviral gene transfer of AdDL, AdWNT5A, AdTGFB and AdDL, AdTGFB and AdWnt5a. Submesothelial zone is indicated by double ended arrow. E, Co‐expression of AdWNT5A and AdTGFB results in a decreased submesothelial thickness (*P* = .001). F‐G, Factor VIII stained sections of mouse peritoneum treated with AdTGFB alone and co‐treatment with AdTGFB and AdWNT5A. Blood vessels are indicated by black arrows. H, TGFB induces an increase in blood vessel density in the peritoneum (*P* = .001). Co‐treatment with AdTGFB and AdWnt5a inhibits this angiogenic response (*P* < .01). I, Representative blot. J, Wnt5a inhibits levels of VEGF in the omentum (*P* = .042). Each bar represents mean ± SD, n = 4

The role of WNT5A in peritoneal membrane injury is poorly understood and limited studies have reported its exact function in fibrotic diseases.^21^, ^22^, ^25^ We co‐expressed TGFB and WNT5A to understand its effect on peritoneal fibrosis. Adenoviral gene transfer of a control adenovirus (AdDL) to the peritoneum produced minimal histologic changes (Figure 1A). The addition of AdWNT5A alone resulted in a slight increase in submesothelial thickness; however, this was not statistically significant (Figure 1B). AdTGFB induced a significant thickening of the submesothelial region, 10 days after infection (Figure 1C). Animals that were co‐treated with AdTGFB and AdWNT5A exhibited a marked reduction in submesothelial thickness (*P* = .001) compared with AdTGFB alone (Figure 1D, quantified in Figure 1E). We also assessed angiogenesis by analysing changes in blood vessel density and VEGF protein. Analysis of blood vessels stained for factor VIII confirmed that AdTGFB induced an increase in blood vessel density in the peritoneum (Figure 1F). Co‐expression of AdTGFB and AdWNT5A inhibited this response as we observed a reduction in blood vessel density (*P* < .01) (Figure 1G, quantified in Figure 1H). AdWNT5A alone did not induce an increase in angiogenesis and appeared similar to control adenovirus (Figure 1H). We also examined the concentration of VEGF protein in the peritoneal tissue by Western blot analysis and observed a decrease in VEGF when AdWNT5A was co‐infected with AdTGFB (*P* = .042) (Figure 1I,J).

### 3.2 WNT5A inhibits EMT in mesothelial cells

**Figure 2:**
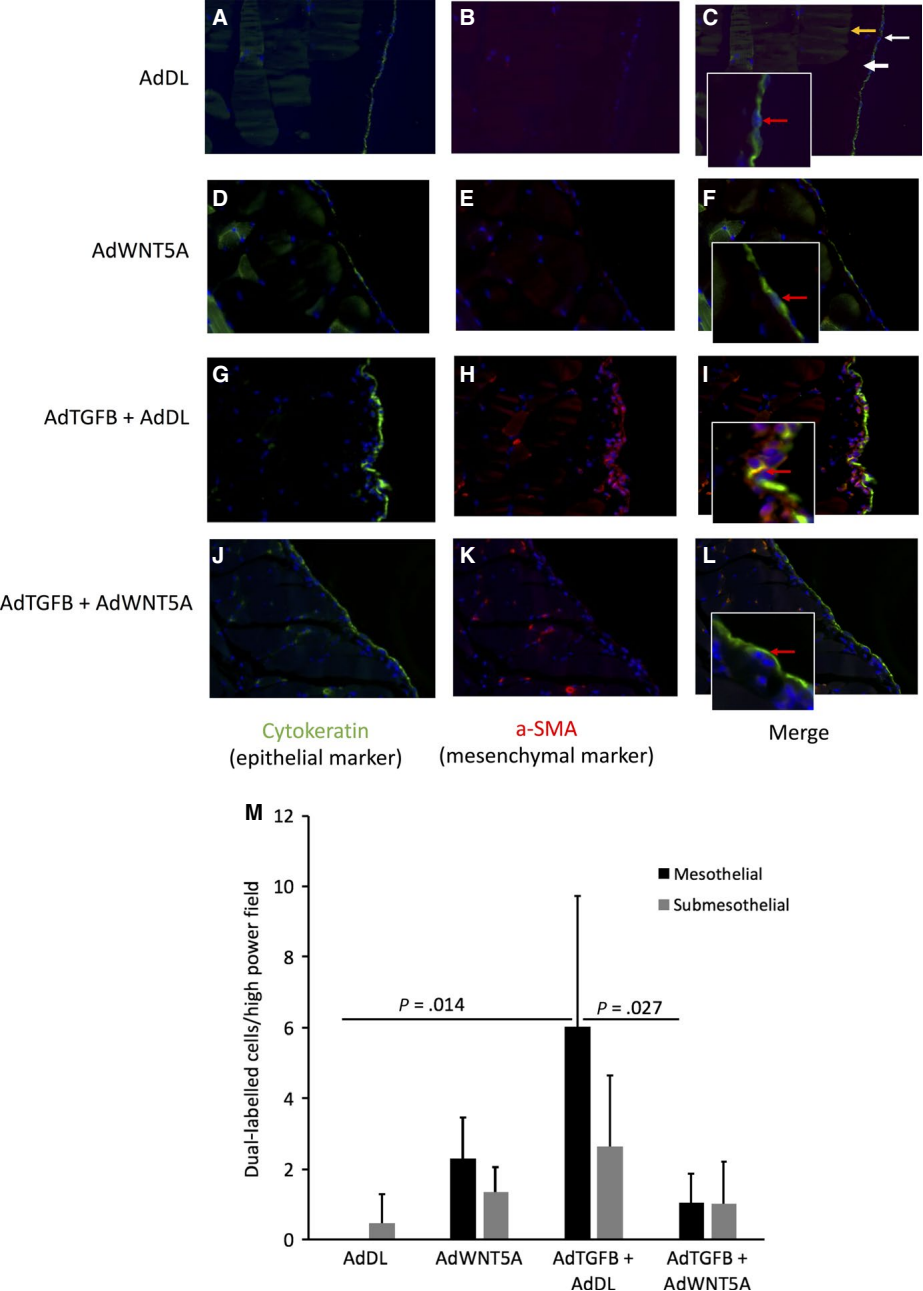
Immunofluorescence for cytokeratin (green), α‐SMA (red) and dapi (blue) in C57Bl/6 mice after adenoviral gene delivery. A‐C, AdDL‐treated mice demonstrate normal peritoneal membrane structure with monolayer of mesothelial cells (thin arrow) expressing cytokeratin and little α‐SMA (red arrow) and a thin submesothelial zone (thick arrow). Underlying this are the abdominal muscles (yellow arrow). D‐F, Mice treated with AdWNT5A also exhibit normal structure similar to controls with mesothelial cells expressing cytokeratin and little α‐SMA (red arrow). G‐I, Infection with AdTGFB increased dual‐labelled cells expressing both cytokeratin and α‐SMA in the mesothelial layer (red arrow). J‐L, Co‐infection with AdTGFB and AdWNT5A resulted in a significant decrease in dual‐labelled cells in the mesothelial layer (red arrow). M, Quantification of dual‐labelled cells demonstrates that WNT5A inhibits mesothelial cell transition (*P* = .027)

WNT/β‐catenin signalling has been reported to interact with the TGFB pathway to promote EMT in different fibrotic diseases with emerging evidence in the peritoneum.^11^, ^14^, ^15^, ^44^ During peritoneal membrane injury, mesothelial cells undergoing EMT lose their epithelial phenotype, down‐regulating characteristic proteins such as cytokeratin and E‐cadherin. Concurrently, these cells rearrange their cytoskeleton becoming more mobile and expressing mesenchymal proteins such as α‐SMA acquiring a myofibroblast phenotype.^45^ We used dual immunofluorescence to identify cells undergoing EMT in the peritoneal membrane. Cytokeratin was used as an epithelial marker and aSMA as a mesenchymal marker (Figure 2A‐M). Cells expressing both cytokeratin and aSMA were marked as dual labelled or cells undergoing EMT. Dual‐labelled cells in the superficial mesothelial location and in the submesothelial zone were quantified (Figure 2M). Mice treated with AdDL exhibited a monolayer of mesothelial cells expressing cytokeratin with few dual‐labelled cells in the mesothelial and submesothelial zone (Figure 2A‐C,M). Mice treated with AdWNT5A exhibited some increase in dual‐labelled cells in both the mesothelial and submesothelial layers; however, this was not statistically significant (Figure 2D‐F,M). AdTGFB infection resulted in a significant increase in cells expressing both cytokeratin and α‐SMA in the mesothelium (*P* = .014) (Figure 2G‐I,M). Co‐treatment with TGFB and WNT5A resulted in suppression of dual‐labelled cells only in the mesothelial layer (*P* = .027) (Figure 2J‐M). Dual‐labelled cells in the submesothelial zone were not significantly affected (Figure 2M).

### 3.3 WNT5A antagonizes WNT/β‐catenin signalling

**Figure 3:**
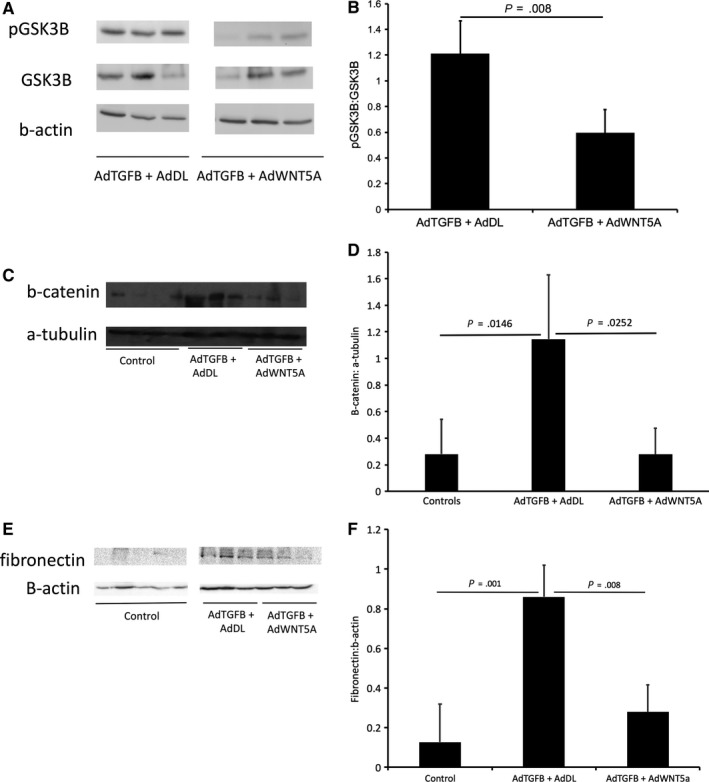
A, Representative blot. B, Western blot analysis revealed a marked decrease in relative pGSK3B levels in response to WNT5A compared with TGFB (*P* = .008). Data presented as mean ± SD, n = 4. C, Representative blot. D, Western blot analysis also demonstrated that TGFB induced a significant increase in total b‐catenin levels (*P* = .146), which is attenuated in response to WNT5A (*P* = .0252). E, Representative blot. F, TGFB induced a significant increase in fibronectin expression (*P* = .001). This was weakened with the addition of WNT5A (*P* = .008). Data presented as mean ± SD, n = 3

We observed that co‐expression of WNT5A and TGFB attenuated fibrosis and angiogenesis suggesting resistance to peritoneal membrane injury. In the setting of wound healing, studies have inconsistently reported WNT5A as either inducing or protecting against injury. Furthermore, some studies have reported the antagonism between β‐catenin dependent and independent signalling. These studies demonstrate that WNT5A activity is dependent on the receptor context. In the presence of FZD, WNT5A activates WNT/β‐catenin and in the presence of Ror2, WNT5A inhibits WNT/β‐catenin.^40^ Activation of WNT/β‐catenin signalling results in phosphorylation of GSK3β and stabilization of β‐catenin levels.^10^ In our study, we investigated the effect of WNT5A on different components of the WNT/β‐catenin signalling pathway. To assess this, we analysed levels of pGSK3β, total β‐catenin and its respective targets when exposed to AdTGFB alone and co‐treatment with AdTGFB and AdWNT5A. We have been suggested that WNT5A is antagonizing this pathway resulting in decreased amounts of pGSK3β and total β‐catenin. Western blot analysis revealed a marked decrease in relative pGSK3B in response to AdTGFB and AdWNT5A compared with AdTGFB alone (*P* = .008) (Figure 3A,B). AdTGFB exposure resulted in an increase in total β‐catenin levels (*P* = .015), and this effect was attenuated with the co‐expression of AdTGFB and AdWNT5A (*P* = .025) (Figure 3C,D). To further confirm a change in b‐catenin transcriptional activity, we also analysed expression of WNT/β‐catenin target proteins in response to AdTGFB and AdWNT5a. Both VEGF and fibronectin have been reported to be target genes of WNT/β‐catenin signalling. As previously mentioned, VEGF protein was significantly decreased in response to AdTGFB and AdWNT5A (*P* = .042) (Figure 1I,J). TGFB induced a significant elevation in fibronectin levels compared to controls (*P* = .001), and levels were reduced after treatment with both AdTGFB and AdWNT5A (*P* = .008) (Figure 3E,F).

### 3.4 TGFB induces WNT5A during peritoneal membrane injury

**Figure 4:**
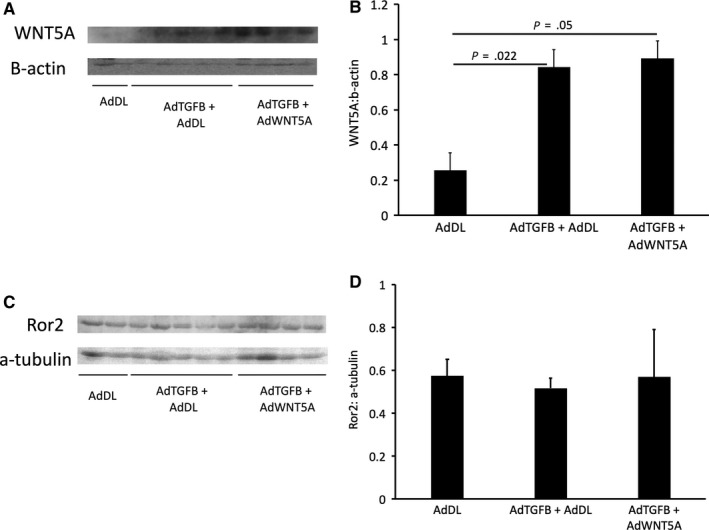
A, Representative blot. B, Western blot analysis of WNT5A demonstrated an increase in WNT5A protein levels in response to TGFB (*P* = .022). Co‐treatment with TGFB and WNT5A also induced a significant increase in WNT5A protein compared to controls (*P* = .05). C, Representative blot. D, Quantitative analysis of Ror2 protein revealed similar levels after TGFB exposure compared to controls. Co‐treatment with TGFB and WNT5A did not significantly alter Ror2 expression. Data presented as mean ± SD, AdDL n = 2, AdTGFB and AdDL n = 5, AdTGFB and AdWNT5A n = 4

In our model of TGFB‐induced peritoneal membrane injury, we wanted to examine the effect of TGFB on both WNT5A and Ror2 protein levels. We observed elevated amounts of WNT5A after treatment with AdTGFB compared to null adenovirus (*P* = .022) (Figure 4A,B). Co‐infection with both AdTGFB and AdWNT5A resulted in a similar increase in WNT5A (*P* = .05) (Figure 4A,B). Ror2 levels appeared similar when exposed to AdTGFB compared to controls. Co‐treatment with AdTGFB and AdWNT5A did not further alter Ror2 expression (Figure 4C,D).

### 3.5 WNT5A is linked to Ror2 in the regulation of fibronectin in human mesothelial cells

**Figure 5:**
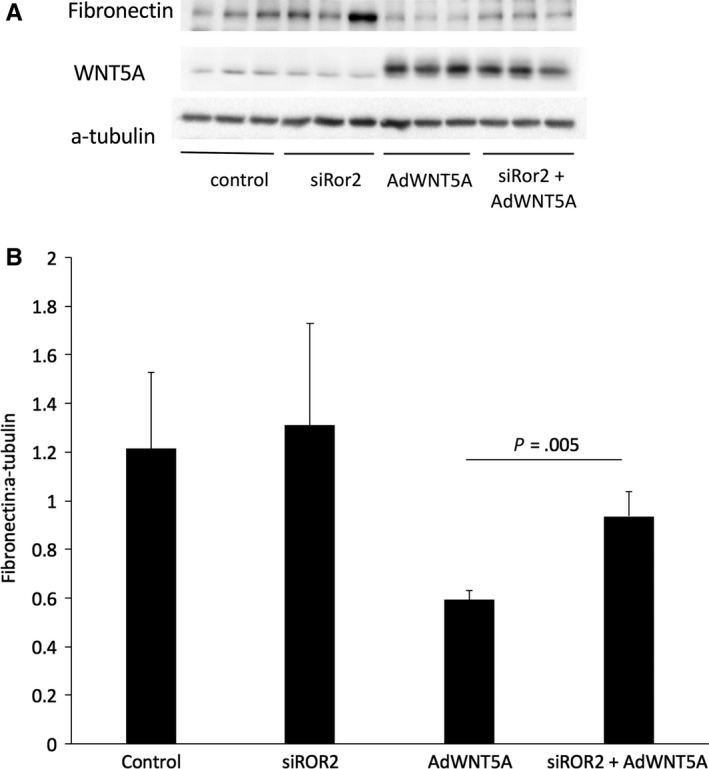
A, Representative blot. B, Western blot analysis revealed an increase in levels of fibronectin when Ror2 was silenced; however, this was not significant. Cells treated with AdWNT5A alone exhibited some decrease in fibronectin expression and blocking Ror2 in AdWNT5A‐treated cells resulted in a significant increase in fibronectin levels (*P* = .005)

We observed that WNT5A inhibits angiogenesis and submesothelial thickening in the presence of Ror2 in this model of peritoneal membrane injury. Existing evidence suggests that WNT5A has differential roles in fibrogenesis based on the receptor context and inhibits WNT/β‐catenin signalling in the presence of Ror2.^40^ We suggest that WNT5A may be signalling through Ror2 to protect against fibrosis. We used a human mesothelial cell line to examine the role of WNT5A Ror2 signalling. MET5A cells were exposed to adenovirus expressing WNT5A and transfected with a siRNA to block Ror2. We confirmed WNT5A expression in cells treated with adenovirus expressing WNT5A (Figure 5A). When independently blocking Ror2, we observed a slight increase in fibronectin levels; however, overall inhibition of Ror2 did not have a large effect. In response to AdWNT5A alone, there was some decrease in fibronectin levels and blocking Ror2 significantly augmented fibronectin expression (*P* = .005).

### 3.6 Ror2 regulates fibrosis and angiogenesis‐related proteins in TGFB‐induced mesothelial cell injury

Our observations suggested that WNT5A and Ror2 work together to regulate levels of fibronectin in human mesothelial cells. Thus, we sought to understand the role of Ror2 in the context of peritoneal membrane injury including fibrosis and angiogenesis. We expanded our investigations using a TGFB model of mesothelial cell injury. We exposed MET5A cells to recombinant TGFB to induce mesothelial cell injury and used an siRNA to silence Ror2. We examined fibronectin and VEGF levels as markers of fibrosis and angiogenesis.

**Figure 6:**
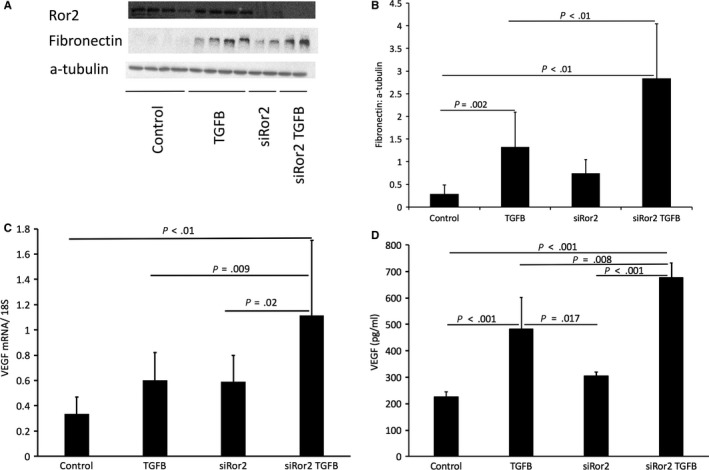
A, Representative blot. B, Western blot analysis of fibronectin demonstrated an increase in response to recombinant TGFB (*P* = .002). Blocking Ror2 exacerbated this effect (*P* < .01) (C) Quantitative analysis of VEGF mRNA: TGFB induced an increase in VEGF mRNA. When using an siRNA to block Ror2, there was a further increase in VEGF mRNA (*P* = .009) (D) ELISA analysis of VEGF protein in cell culture supernatant. TGFB induced an increase in VEGF protein levels (*P* < .001). When silencing Ror2, there was an even greater increase in VEGF protein (*P* = .008). Data presented as mean ± SD, n = 6

Ror2 was successfully silenced (Figure 6A), and WNT5A expression was confirmed in MET5A cells (Figure S1). TGFB induced an increase in fibronectin (*P* = .002), and this effect was amplified with Ror2 silencing (*P* < .01) (Figure 6A,B). We also observed increased levels of VEGF mRNA in response to TGFB and blocking Ror2 resulted in an augmentation of VEGF mRNA (*P* = .009) (Figure 6C). Using ELISA analysis, we assessed levels of VEGF protein in supernatant from cultured mesothelial cells. TGFB induced an increase in VEGF protein (*P* < .001), and this effect was exacerbated in response to Ror2 silencing (*P* = .008) (Figure 6D).

## 4 DISCUSSION

Patients undergoing peritoneal dialysis begin to develop a change in peritoneal membrane structure including fibrosis and angiogenesis. Peritoneal fibrosis has been associated with a decline in ultrafiltration capacity by altering hydraulic conductance of the membrane observed in many biopsy studies.^4^, ^46^ Expansion of peritoneal vasculature is also suggested to be associated with increased solute transport or leakiness of the membrane.^6^ High solute transport results in increased reabsorption of glucose and loss of the solute gradient that drives ultrafiltration.^3^ Although fibrosis and angiogenesis are interrelated events, angiogenesis, and the associated increased solute transport is associated with worse patient outcome and is therefore an important therapeutic target.^4^

In this study, we assessed the role of WNT5A, in the development of both fibrosis and angiogenesis and observed that it reduces both elements of peritoneal membrane injury. Addition of WNT5A to the mouse peritoneum resulted in reduced submesothelial thickness and blood vessel density in TGFB‐induced peritoneal fibrosis. Accumulating evidence has shown that WNT5A exhibits distinct effects depending on the cell surface receptor present. Studies of angiogenesis report that WNT5A can either stimulate^47^ or inhibit endothelial cells^48^ and its effect may be attributed to receptor context. Emerging functional studies in fibrosis have suggested a more deleterious role for WNT5A in fibrosis,^23^, ^26^ and most have shown WNT5A to exacerbate injury in the presence of FZD receptors.^31^ For instance, in a study of asthma development WNT5A and TGFB resulted in ECM production in airway smooth muscle cells and these cells demonstrated high levels of FZD8. In conjunction with WNT5A, this WNT receptor was observed to regulate TGFB‐induced expression of collagen and fibronectin. Minimal levels of Ror2 expression were observed in these cells, and Ror2 was not further investigated.^29^ In other studies, WNT5A has been observed to control cell migration through FZD1 and 2.^49^ In our study, WNT5A alone seemed to have a small effect in increasing submesothelial thickening and EMT. Interestingly co‐expression of WNT5A with TGFB reduced submesothelial thickening and angiogenesis as well as EMT molecular markers in mesothelial cells. Here, we confirmed Ror2 expression and observed that these changes occurred in the presence of Ror2. We suggest that these effects may be attributed to a change in receptor environment during injury. Further, WNT5A may play a bidirectional role in peritoneal membrane injury depending on which receptors are present. As levels of Ror2 remained unchanged, it is possible that other receptors are contributing to the effects of WNT5A during TGFB injury and it may be a specific combination of receptors which promote protection. Ultimately, future investigations examining the receptor environment are necessary.

We further examined the function of this receptor in injured mesothelial cells by silencing Ror2. With the addition of AdWNT5A, we observed an increase in levels of fibronectin when Ror2 was inhibited. We expanded our investigation to examine a TGFB model of mesothelial cell injury. Overexpression of TGFB induced an increase in fibronectin and VEGF. Interestingly when Ror2 was silenced, increases in fibronectin and VEGF protein were observed. This suggests that Ror2 potentially has some protective capacity in injured mesothelial cells by regulating fibrotic and angiogenic factors.

In contrast to our observations, WNT5A and Ror2 were shown to contribute to injury in a recent study of renal fibrosis.^30^ WNT5A and Ror2 were both elevated after unilateral ureteral obstruction (UUO), and Ror2 expression was associated with expression of EMT‐related proteins. Ror2 heterozygotes were generated to partially reduce Ror2 and exposed to UUO, but did not yield altered levels of EMT markers. However, lower levels of Ror2 did result in reduced damage to the basement membrane.^30^ Although this study suggests that Ror2 is contributing to injury, it is unclear how this receptor is functioning in the context of WNT5A. It may be acting as a co‐receptor alongside FZD and stimulating a different pathway. Moreover, Ror2 heterozygotes still express Ror2 to some degree. Interestingly, Ror2 heterozygotes showed an increase in Dvl2 which is a component of both canonical and non‐canonical WNT signalling. In our study, we directly co‐expressed WNT5A with TGFB and observed a reduction in angiogenesis and fibrosis. TGFB‐induced WNT5A expression, however, did not substantially affect Ror2 receptor levels. In vitro, we examined the link between WNT5A and Ror2 more closely. We overexpressed WNT5A and used an siRNA to silence Ror2, and we observed that WNT5A and Ror2 are involved in down‐regulating fibronectin. We observed a similar result in a TGFB model of human mesothelial cell injury where absence of Ror2 resulted in increases in fibronectin and VEGF. In the future, studies that outline the mechanism by which Ror2 is acting and which downstream pathways it may be activating or inhibiting during the development of injury will shed more light on WNT5A/Ror2 signalling. Moreover, several transcription factors regulate the expression of WNT5A, which is different in each cell type; thus, surrounding signalling pathways require closer analysis.^31^

It has become apparent that WNT5A can also regulate β‐catenin dependent signalling. Mikels and colleagues, observed the dual nature of WNT5A where it could both activate and inhibit the canonical WNT/β‐catenin signalling pathway depending on the cell surface receptor that was present. In the presence of FZD4, WNT5A increased β‐catenin levels. Paradoxically, in the presence of Ror2, WNT5A suppressed β‐catenin activity in vitro.^40^ Therefore, WNT5A can lead to activation of two discrete pathways based on receptor context and these observations were validated in other studies.^50^ Levels of β‐catenin are controlled by a complex of proteins including GSK3B, which marks β‐catenin for ubiquitination and degradation. When WNT signalling is turned on, GSK3B is phosphorylated and inactivated resulting in stabilization of β‐catenin and up‐regulation of its target genes. We found WNT5A induced a decrease in both pGSK3B, total b‐catenin levels and target protein fibronectin and therefore may be implicated in antagonizing WNT/β‐catenin signalling. Our results agree with other studies where WNT5A has inhibited canonical WNT signalling in the development of cancer.^51^ Other groups have reported that WNT5A blocks transcriptional activity of β‐catenin and does not directly impact levels of pGSK3B or β‐catenin protein levels.^40^, ^52^, ^53^ Our results demonstrate a more upstream effect as we observed WNT5A to reduce levels of pGSK3B, which coincided with decreased levels of total b‐catenin. The molecular mechanism by which WNT5A regulates WNT/β‐catenin signalling requires further investigation. As well, the receptor environment requires closer examination.

The peritoneal membrane is a lifeline for patients with end‐stage kidney disease on peritoneal dialysis therapy. We know that injury to the peritoneum increases the risk of technique failure and mortality. This current research expands potential therapeutic options for preservation of the peritoneal membrane in PD patients. Based on our findings, we suggest that WNT5A blocks the canonical WNT pathway to reduce both fibrosis and angiogenesis in the peritoneum and Ror2 is a potential candidate for mediating these effects. Although further investigation is necessary, the dual nature of WNT5A may be used as a method of targeting and blocking aberrant WNT/β‐catenin signalling to prevent progression of injury.

## CONFLICT OF INTEREST

No conflicts of interest, financial or otherwise, are declared by the authors.

## AUTHOR CONTRIBUTIONS

MP and PJM conceived and designed research, analysed data, interpreted results of experiments, prepared figures, edited and revised manuscript, and approved final version of manuscript; MP and LL performed experiments; and MP drafted manuscript.

## Data Availability

The data that support the findings of this study are available from the corresponding author upon reasonable request.
