# Supplementary figures and images for: The role of WNT5A and Ror2 in peritoneal membrane injury
Source: J Cell Mol Med. 2020 Feb 13;24(6):3481–91. doi: 10.1111/jcmm.15034 (PMC7131918; doi:10.1111/jcmm.15034)

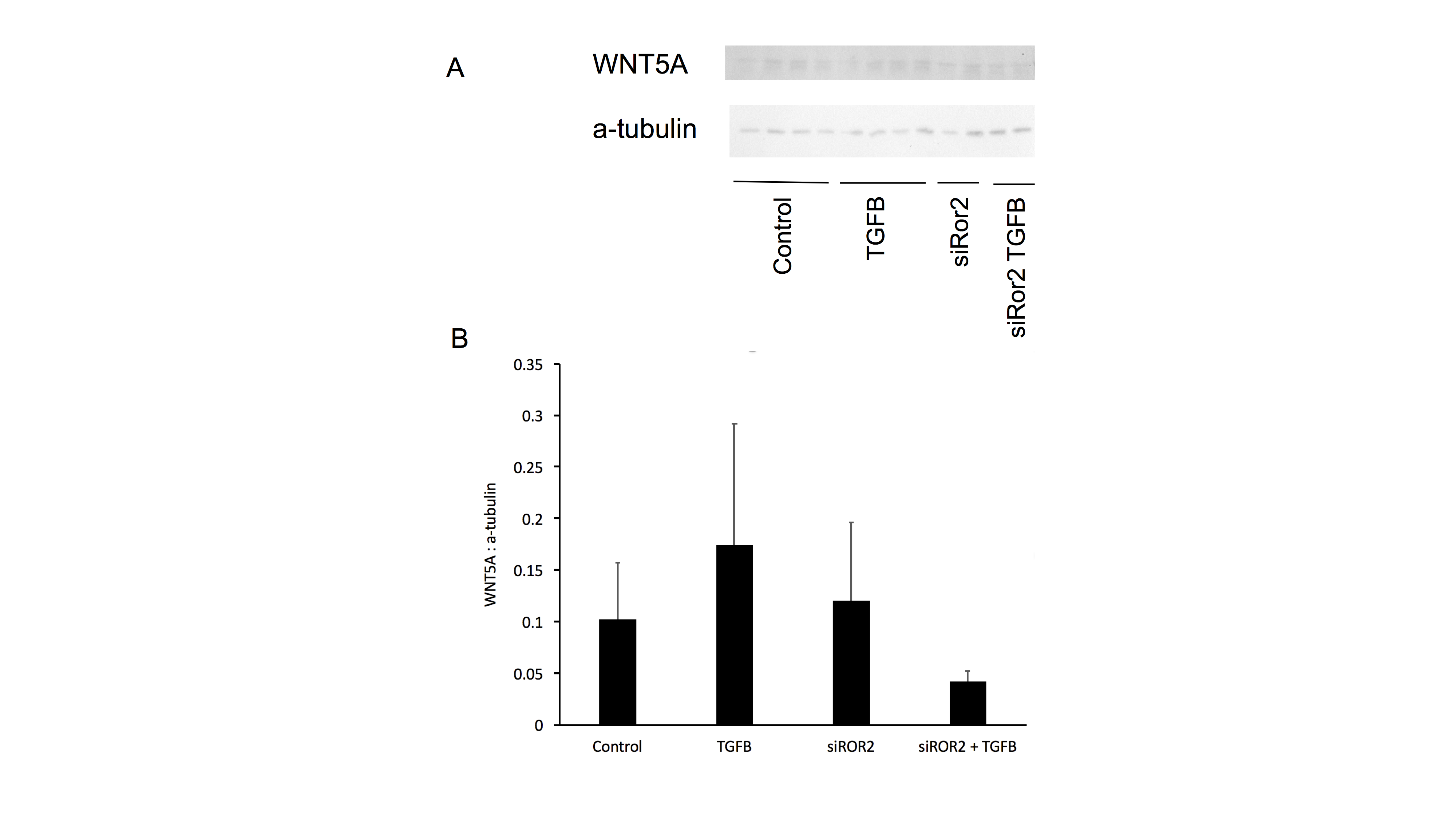

Supplement: Supplementary file 1 [file JCMM-24-3481-s001.tiff]
